# Bloom’s Syndrome and PICH Helicases Cooperate with Topoisomerase IIα in Centromere Disjunction before Anaphase

**DOI:** 10.1371/journal.pone.0033905

**Published:** 2012-04-26

**Authors:** Sébastien Rouzeau, Fabrice P. Cordelières, Géraldine Buhagiar-Labarchède, Ilse Hurbain, Rosine Onclercq-Delic, Simon Gemble, Laura Magnaghi-Jaulin, Christian Jaulin, Mounira Amor-Guéret

**Affiliations:** 1 Institut Curie, Centre de Recherche, Centre Universitaire, Bât, Orsay, France; 2 CNRS UMR 3348, Stress Génotoxiques et Cancer, Orsay, France; 3 Institut Curie, Centre de Recherche, Plateforme d’Imagerie Cellulaire et Tissulaire IBiSA, Centre Universitaire, Bât, Orsay, France; 4 Institut Curie, Centre de Recherche, Paris, France; 5 Structure et Compartimentation membranaire, CNRS UMR144, Paris, France; 6 Plateforme d’Imagerie Cellulaire et Tissulaire IBiSA, CNRS UMR 144, Paris, France; 7 Institut de Génétique et Développement, CNRS UMR 6290, Université de Rennes 1, Rennes, France; Duke University, United States of America

## Abstract

Centromeres are specialized chromosome domains that control chromosome segregation during mitosis, but little is known about the mechanisms underlying the maintenance of their integrity. Centromeric ultrafine anaphase bridges are physiological DNA structures thought to contain unresolved DNA catenations between the centromeres separating during anaphase. BLM and PICH helicases colocalize at these ultrafine anaphase bridges and promote their resolution. As PICH is detectable at centromeres from prometaphase onwards, we hypothesized that BLM might also be located at centromeres and that the two proteins might cooperate to resolve DNA catenations before the onset of anaphase. Using immunofluorescence analyses, we demonstrated the recruitment of BLM to centromeres from G2 phase to mitosis. With a combination of fluorescence *in situ* hybridization, electron microscopy, RNA interference, chromosome spreads and chromatin immunoprecipitation, we showed that both BLM-deficient and PICH-deficient prometaphase cells displayed changes in centromere structure. These cells also had a higher frequency of centromeric non disjunction in the absence of cohesin, suggesting the persistence of catenations. Both proteins were required for the correct recruitment to the centromere of active topoisomerase IIα, an enzyme specialized in the catenation/decatenation process. These observations reveal the existence of a functional relationship between BLM, PICH and topoisomerase IIα in the centromere decatenation process. They indicate that the higher frequency of centromeric ultrafine anaphase bridges in BLM-deficient cells and in cells treated with topoisomerase IIα inhibitors is probably due not only to unresolved physiological ultrafine anaphase bridges, but also to newly formed ultrafine anaphase bridges. We suggest that BLM and PICH cooperate in rendering centromeric catenates accessible to topoisomerase IIα, thereby facilitating correct centromere disjunction and preventing the formation of supernumerary centromeric ultrafine anaphase bridges.

## Introduction

The centromere is a highly differentiated chromosomal structure consisting, in human cells, of α-satellite DNA repeats [1]. It plays an essential role in cell division, particularly in kinetochore assembly, and in ensuring the segregation of equal number of chromosomes to daughter cells during mitosis [2]. The maintenance of centromere stability is thus essential to prevent chromosomal instability and cancer development [3]. Our interest in centromere stability began with the unexpected discovery that a helicase-like protein, PICH (Plk1-interacting checkpoint “helicase”) and the Bloom syndrome helicase (BLM) colocalized to centromeric ultrathin DNA threads that could not be counterstained with conventional DNA dyes or antibodies against histones [4], [5]. These threads were found to be common in all cultured normal cells tested, and are therefore probably physiological structures. These PICH- and BLM-positive DNA threads, which are also called ultrafine anaphase bridges (UFBs), are thought to contain unresolved DNA catenations between the centromeres separating during anaphase or to originate from incompletely replicated DNA [4], [5]. Almost all the UFBs detected in untreated cells are of centromeric origin and have been proposed to prevent reactivation of the spindle assembly checkpoint (SAC) during early anaphase, by maintaining tension across centromeres [4], [6]. As cells progress through anaphase, UFBs become progressively longer and decrease in number. They are no longer detectable by telophase. The resolution of centromeric UFBs requires topoisomerase IIα (Topo IIα) activity and occurs after the onset of anaphase, after the disappearance of cohesin [6], [7]. Thus, sister centromeres are held together through double-stranded DNA catenations until the end of anaphase, accounting for the strong induction of centromeric UFBs by catalytic inhibitors of Topo II [4], [5]. The frequency of PICH-positive UFBs is also higher in BLM-deficient cells than in control cells, suggesting the involvement of BLM in their resolution [5]. This point is of particular interest because BLM deficiency causes Bloom’s syndrome, an autosomal recessive disease displaying one of the strongest known correlations between chromosomal instability and an increase in the risk of cancer at an early age [8]. The hallmark of BLM-deficient cells is a high frequency of sister chromatid exchanges (SCEs) [8]. Thus, BLM plays a crucial role in preventing genetic instability and cancer. In normal cells, BLM is detected only on UFBs in anaphase, whereas PICH staining is detected as early as metaphase [5]. PICH is required for the localization of BLM to anaphase UFBs, and BLM is required for the chromatin remodeling function of PICH *in vivo*, the two proteins cooperating to limit histone incorporation into UFBs and to promote their resolution [9]. We hypothesized that the functional relationship between PICH and BLM might also be of major importance before anaphase in the maintenance of centromere integrity.

We report here the localization of BLM to the centromere of chromosomes from G2 phase to mitosis. We show that PICH and BLM deficiencies are associated with changes in centromere structure, with an increase in centromeric non disjunction in cohesin-depleted cells and a defect in the recruitment of active Topo IIα to centromeres. Our results reveal the existence of a new centromeric mechanism involving cooperation between PICH and BLM, likely to render some centromeric catenates accessible to Topo IIα before anaphase onset, thereby facilitating correct centromeric disjunction and preventing the formation of supernumerary UFBs.

## Results

### BLM is a Centromeric Protein

**Figure 1:**
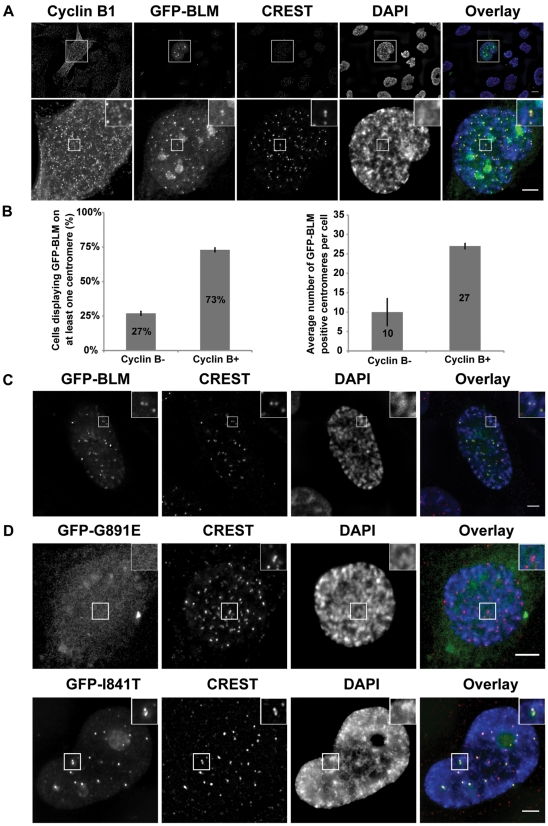
GFP-BLM localizes to centromeres in G2/prophase cells. (A) Localization of GFP-BLM (green) to centromeres in late G2 cells. Nuclei were visualized by DAPI staining (blue). G2 cells were stained with antibodies against cyclin B1. Cells were stained with CREST serum to visualize centromeres (red). Lower panels are the magnification of the corresponding upper panels. Scale bar = 5 µm. (B) Two hundred cells with GFP-BLM-positive centromeres were analyzed by staining for cyclin B1 (cyclin B- or cyclin B+). The percentage of cells in each category is indicated (left panel), together with the mean number of GFP-BLM-positive centromeres per cell (right panel). Bars indicate the standard deviation (SD). (C) Localization of GFP-BLM (green) to centromeres (red) in prophase cells. The nucleus and centromeres were visualized as in A. Scale bar = 5 µm. (D) Localization of GFP-BLM (green) with an inactive helicase domain (GFP-I841T) or with both the helicase and DNA-binding domains inactivated (GFP-G891E). Nuclei and centromeres were visualized as in A. Scale bar = 5 µm.

PICH is a centromeric protein [4]. As BLM, like PICH, localized with centromeric UFBs [5], we first investigated whether BLM also localized to centromeres. We analyzed the subcellular distribution of stably expressed GFP-tagged BLM protein in asynchronous GM08505 BS cells (GFP-BLM cells) [10]. We detected GFP-BLM in PML bodies, as expected [11] (Figure S1), but in some cells, BLM foci were smaller, double-dotted and no longer colocalized within PML bodies. Using CREST serum as a centromeric marker, we showed, by cyclin B1 staining [12], that 73% of cells with GFP-BLM-positive centromeres were in G2-phase or early mitosis (Figure 1A and 1B, left panel). These cells included a mean of 27 GFP-BLM-positive pairs of centromeres (7 to 59 pairs). By contrast, only 10 GFP-BLM-positive centromeres (1 to 24 pairs) were detected in late S-phase cells (most centromeres being paired), negative for cyclin B1 staining (Figure 1B, right panel). All the centromeres in prophase cells displayed positive staining for GFP-BLM (Figure 1C). These results suggest that BLM is progressively loaded to the centromeres from late S phase until mitosis. We then investigated whether the helicase and/or DNA binding activities of BLM were required for centromeric localization, using GM08505 BS cells stably transfected with a construct encoding either the BLM protein with an inactive helicase domain (GFP-I841T) or with both the helicase and DNA binding domains inactivated (GFP-G891E) [13]. We found that DNA-binding activity was required for the centromeric localization of BLM, whereas helicase activity was not. Indeed, GFP-G891E displayed a diffuse distribution in BS cells and was not detectable at centromeres, suggesting that BLM may interact with centromeric DNA (Figure 1D).

**Figure 2:**
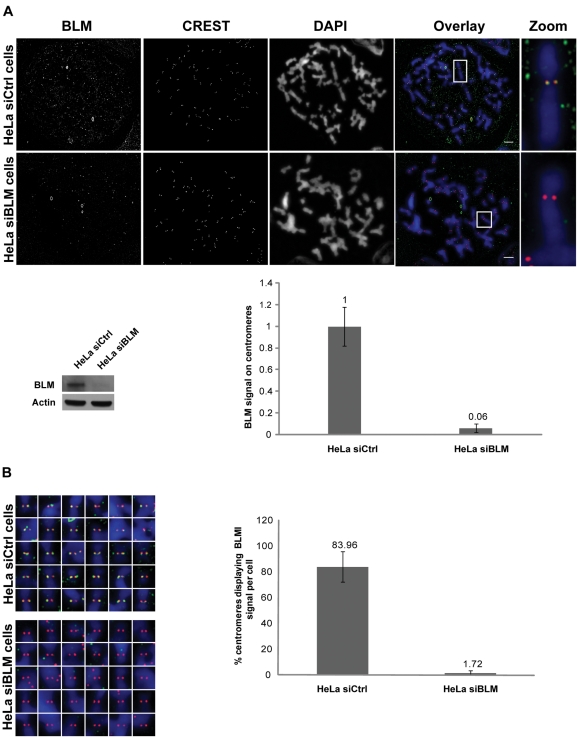
BLM localizes to centromeres in mitotic cells. (A) Endogenous BLM localizes to centromeres in HeLa cells. Wide-field microscopy after immunofluorescence staining on chromosome spreads obtained by cytocentrifugation of BLM siRNA-transfected or control siRNA-transfected HeLa S3 cells. Staining is shown for BLM (green), CREST (red) and chromosomes (blue). Single chromosome magnifications are shown (upper panels). Proteins levels were assessed by immunoblotting, probing the same membrane with anti-BLM (ab-476) antibody and then with anti-β actin antibody, as a loading control (lower left panel). Quantification of centromeric fluorescence signals for BLM (normalized according to the CREST signal) for a total of 20 centromeres in control cells (siCtrl) and 24 centromeres in BLM-depleted cells (siBLM) from two independent experiments, demonstrate the specificity of ab476 BLM antibodies (lower right panel). Data are means and SD normalized with respect to controls. Scale bar = 5 µm. (B) Centromeres detected with CREST serum (red) from all the chromosomes of HeLa siCtrl cells and HeLa siBLM cells from Figure 2A were analyzed for BLM signals (green). Chromosomes were visualized by DAPI staining (blue) (left panels). The same analysis was performed on a total of 9 HeLa siCtrl cells and 9 HeLa siBLM cells from two independent experiments: the percentage of centromeres giving a BLM signal is shown (right panel).

The centromeric localization of endogenous BLM was also investigated in immunofluorescence experiments with two different anti-BLM antibodies, on mitotic chromosome spreads from HeLa S3 cells at prometaphase/metaphase stage, downregulated for BLM (siBLM) or without BLM downregulation (siCtrl). In HeLa siCtrl cells, we detected endogenous BLM at centromeres, with CREST serum used as a centromeric marker. The siRNA-mediated depletion of BLM (siBLM) resulted in the absence of the BLM signal at centromeres, confirming the specificity of the anti-BLM antibodies (Figure 2A). Closer examination of the centromeres of each chromosome showed the BLM signal to be present on about 84% of centromeres in HeLa siCtrl cells and on 1.7% of centromeres from HeLa siBLM cells (Figure 2B). Thus, BLM is clearly a centromeric protein.

### BLM and PICH Deficiencies are Associated with Changes in Centromeric DNA Structure

**Figure 3:**
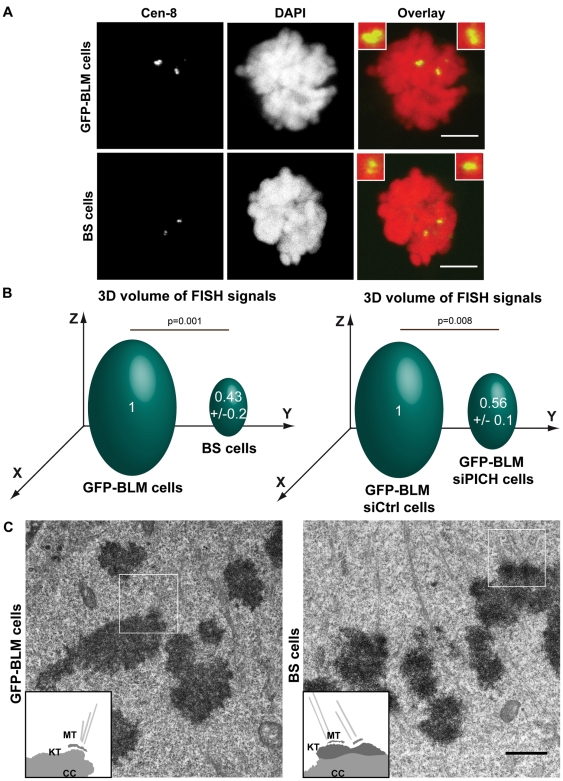
Structural defects at the centromeres in BLM-and PICH-deficient cells. (A) FISH with the CEN-8 probe (green) on metaphase BS and GFP-BLM cells. Chromosomes are visualized by DAPI staining (red). Bar = 5 µm. (B) Comparison of the volume of the centromeric FISH signal detected on chromosomes 8 from GFP-BLM (defined as 1) and BS cells (left panel) and from GFP-BLM cells with (siPICH) and without (siCtrl) PICH downregulation (defined as 1) (right panel). We analyzed 45 metaphase cells from three independent experiments for each cell line. (C) GFP-BLM cells (left panel) and BS cells (right panel) were processed for electron microscopy. Scale bar = 1 µm. Inset: schematic diagram of the regions of interest (white squares) (MT: microtubules, KT: kinetochore outer plate and CC: centromeric chromatin). Eight centromeres from two independent experiments were analyzed.

**Figure 4:**
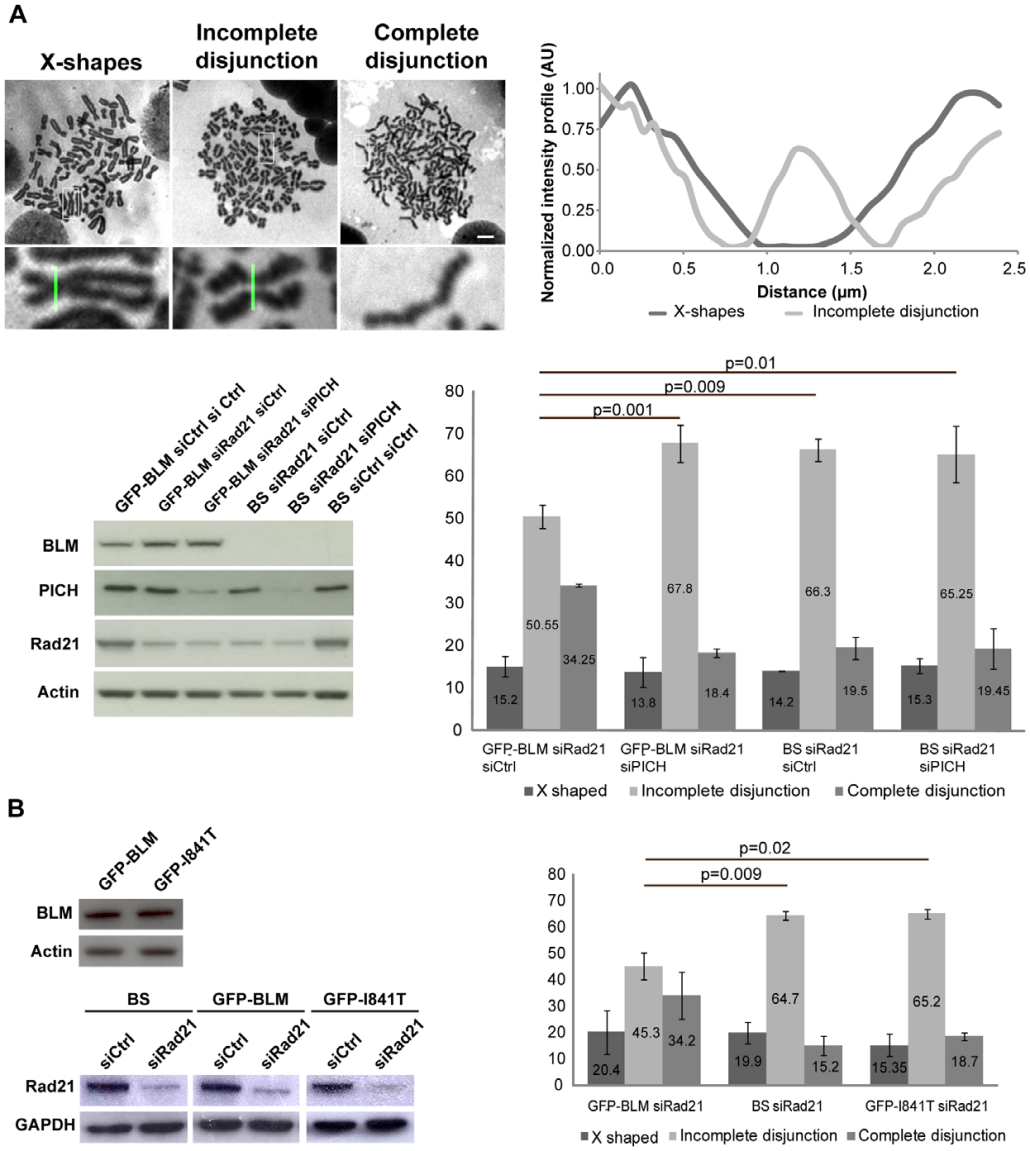
BLM-deficient cells and PICH-downregulated display non disjunction of centromeres and impaired recruitment of active Topo IIα to centromeres. (A) GFP-BLM and BS cells were transfected for 72 hours with Rad21 siRNAs and transfected either with control siRNAs or with PICH siRNAs. BLM, PICH and Rad21 proteins levels were assessed by immunoblotting, probing the same membrane with anti-BLM (ab-476), anti-PICH and anti-Rad21 antibodies and with anti-β actin antibody, as a loading control (lower left panel). Chromosome spreads were performed and sorted on the basis of their phenotype: X-shapes, incomplete disjunction or complete disjunction. The scale bars indicate 5 µm (upper left panel). This classification is based on the intensity profiles of centromeres (upper right panel). We analyzed 500 spreads from three independent experiments for each cell line. The frequency of each phenotype, in each of the three cell lines, is shown in the histogram (lower right panel). Bars represent SD. (B) GFP-BLM, BS and GFP-I841T cells were transfected for 72 hours with Rad21 siRNAs and the same experiments as in (A) (right panels) were carried out. We checked the levels of BLM and Rad21 proteins by western blotting (left panels).

We investigated the possible effects of BLM deficiency on centromere structure. We used a FISH (fluorescence *in situ* hybridization)-based assay to investigate the centromeric DNA organization of one chromosome and its volume (chromosome 8, CEN-8 probe [14]) in GM08505 BLM-deficient cells stably expressing a GFP vector (BS cells) and in GFP-BLM cells during prometaphase and metaphase (Figure 3A). We found that CEN-8 signal volume in BS cells was less than half that in control cells, indicating that BLM is involved in centromeric DNA structure (Figure 3B, left panel). For confirmation of these findings, we analyzed the effects of BLM deficiency on the structural morphology of the centromeric DNA in BS and GFP-BLM metaphase cells by electron microscopy (EM) (Figure 3C). Centromeric chromatin was identified adjacent to the typical kinetochore structure. Immunofluorescence analysis of several kinetochore/centromere markers confirmed that BLM deficiency had no major effect on kinetochore structure, as previously shown for PICH-deficient cells [4] (Figure S2). However, the centromeric chromatin was clearly more dense in BS cells than in GFP-BLM cells (Figure 3C), confirming an effect on centromeric DNA organization of the absence of BLM. We next performed FISH experiments in GFP-BLM cells downregulated for PICH (siPICH) or without PICH downregulation (siCtrl). We found that CEN-8 signal volume in PICH-downregulated cells was about half that in control cells, indicating that PICH is also involved in centromeric DNA structure before anaphase onset (Figure 3B, right panel). These results are not the consequence of a coregulation of *PICH* and *BLM* genes, because the deficiency of one protein did not affect the expression of the other (Figure 4A, lower left panel). Finally, CEN-8 signal volume in HeLa cells depleted of BLM, PICH or both proteins was significantly lower than that in control cells (Figure S3). There was therefore no additive or synergistic effect of the depletion of BLM and PICH on CEN-8 signal volume, suggesting that these proteins are involved in the same regulatory pathway. These results also confirmed the involvment of BLM and PICH in centromeric DNA structure before anaphase onset.

### BLM and PICH are Required for Complete Centromere Disjunction

As BLM localizes to centromeres (Figures 1 and 2), we investigated whether, in addition to its potential role in resolving UFBs during anaphase [5], [15], BLM might be involved in preventing UFB formation, by contributing to the centromeric DNA decatenation process before the metaphase-anaphase transition. DNA catenation induced by the inhibition of Topo IIα has been shown to maintain sister chromatid cohesion in the absence of cohesin complexes [7], [16]. We thus analyzed chromosome spreads from BS cells and from GFP-BLM cells arrested in prometaphase (+colchicine), with (siPICH) or without (siCtrl) PICH knockdown (Figure 4A). For these experiments, Rad21, the cleavable subunit of cohesin, was depleted from all cell lines (siRad21) (Figure 4A, lower left panel). BLM deficiency and PICH knockdown were associated with an increase in centromeric cohesion. Indeed, we observed three distinct and different phenotypes: classical X-shaped chromosomes probably corresponding to cells not transfected with Rad21 siRNA (X-shapes), the expected fully disjoined chromatids resulting from cohesin depletion (complete disjunction), and a third, unusual phenotype of separated sister chromatids that were still physically linked, reflecting incomplete chromatid disjunction (incomplete disjunction) (Figure 4A, upper left panels). Careful examination of these “separated but still paired” chromatids revealed that they were mostly linked via their centromeres (visualized as the major chromosomal constriction). This observation was confirmed by quantifying the intensity profiles of the centromeric region between the two chromatids of the “X-shape” and “incomplete disjunction” phenotypes (Figure 4A, upper right panel). We determined the frequency of each phenotype in the four cell lines and found that about 15% of the prometaphase cells presented the X-shaped phenotype. About 19% of siRad21-treated BS cells, siRad21+siPICH treated GFP-BLM cells, and siRad21+siPICH-treated BS cells presented completely disjoined chromatids, whereas about 66% of these cells, displayed an incomplete centromere disjunction phenotype (Figure 4A, lower right panel). By contrast, in GFP-BLM cells with Rad21 knockdown only, 34.2% of the cells displayed complete sister chromatid disjunction and 50.5% had separated sister centromeres that remained paired (as opposed to the 66% observed in the other cell lines). Similar results were obtained in Rad21-depleted HeLa cells, with a significantly higher frequency of cells presenting an “incomplete disjunction” phenotype and a significantly lower frequency of cells presenting a “complete disjunction” phenotype in cells depleted of BLM or PICH (Figure S4). Rad21 levels were initially similar in GFP-BLM cells and in HeLa cells and siRNA-mediated Rad21 downregulation was similarly effective in all cell lines. The differences in the percentage of cells with an incomplete disjunction phenotype therefore did not reflect differences in the efficacy of siRNA-mediated Rad21 downregulation (Figure S5). Finally, we performed the same chromosome spread analysis with GFP-BLM-I841T cells (inactive helicase domain), comparing the results with those for BS and GFP-BLM cells. Similar results were obtained with both Rad21-depleted GFP-BLM-I841T cells and Rad21-depleted BS cells, (Fig. 4B right panel), despite GFP-BLM-I841T and GFP-BLM cells having similar amounts of BLM protein (Figure 4B, left panel).

Together, these results show that both BLM and PICH are involved in the complete disjunction of sister centromeres independently of the cohesin pathway, strongly suggesting that in the absence of BLM and/or PICH, some centromeric catenations are not processed, leading to an increase in centromeric non disjunction. The results for cells expressing BLM with an inactive helicase domain indicate that the helicase activity of BLM is involved in the complete disjunction of sister centromeres.

Lastly, we observed no additive or synergistic effect of PICH and BLM deficiencies on the “incomplete disjunction” phenotype, suggesting that these two proteins are involved in the same regulatory pathway.

### Centromeric UFBs are not Detectable in PICH-depleted Cells

The findings reported above suggest that the higher frequency of centromeric UFBs in BLM-deficient cells [5], which we also confirmed, may be due to a lack of BLM processing activity of some centromeric DNA catenations. These results also suggest that the centromeric DNA catenations persisting in PICH-deficient cells probably result in an increase in centromere UFB frequency. We investigated the effect of PICH knockdown on the frequency of UFBs, by monitoring BLM. We found that PICH depletion resulted in a three-fold decrease in the number of cells presenting UFBs and in a five-fold decrease in the number of BLM-positive UFBs (Figure S6A and S6B). However, most of the BLM-positive UFBs detected were also positive for PICH staining, indicating that PICH downregulation was incomplete, with UFBs positive for both BLM and PICH probably forming in cells not efficiently transfected with PICH siRNA. Only a minor fraction of UFBs (5–6%) were positive for BLM staining and negative for PICH staining and the frequency of these UFBs was not affected by PICH downregulation (Figure S6B). The lack of detection of an increase in the frequency of BLM-positive UFBs in PICH-depleted anaphase cells can be accounted for by recent observations showing that PICH is required for BLM localization to anaphase threads [15]. Nevertheless, we cannot exclude the possibility that UFBs may be prematurely disrupted in the absence of PICH, as previously suggested [4], or that they may not form at all. We were unable to visualize UFBs in the absence of PICH, even by BrdU staining, probably due to the inherent limitations of this method [17]. However, as PICH downregulation is associated with an increase in the frequency of cells presenting an “incomplete disjunction” phenotype, UFB frequency is probably higher in these cells, although these structures are not detectable in the absence of PICH.

### BLM and PICH are Required for the Recruitment of Topo IIα to Centromeres

**Figure 5:**
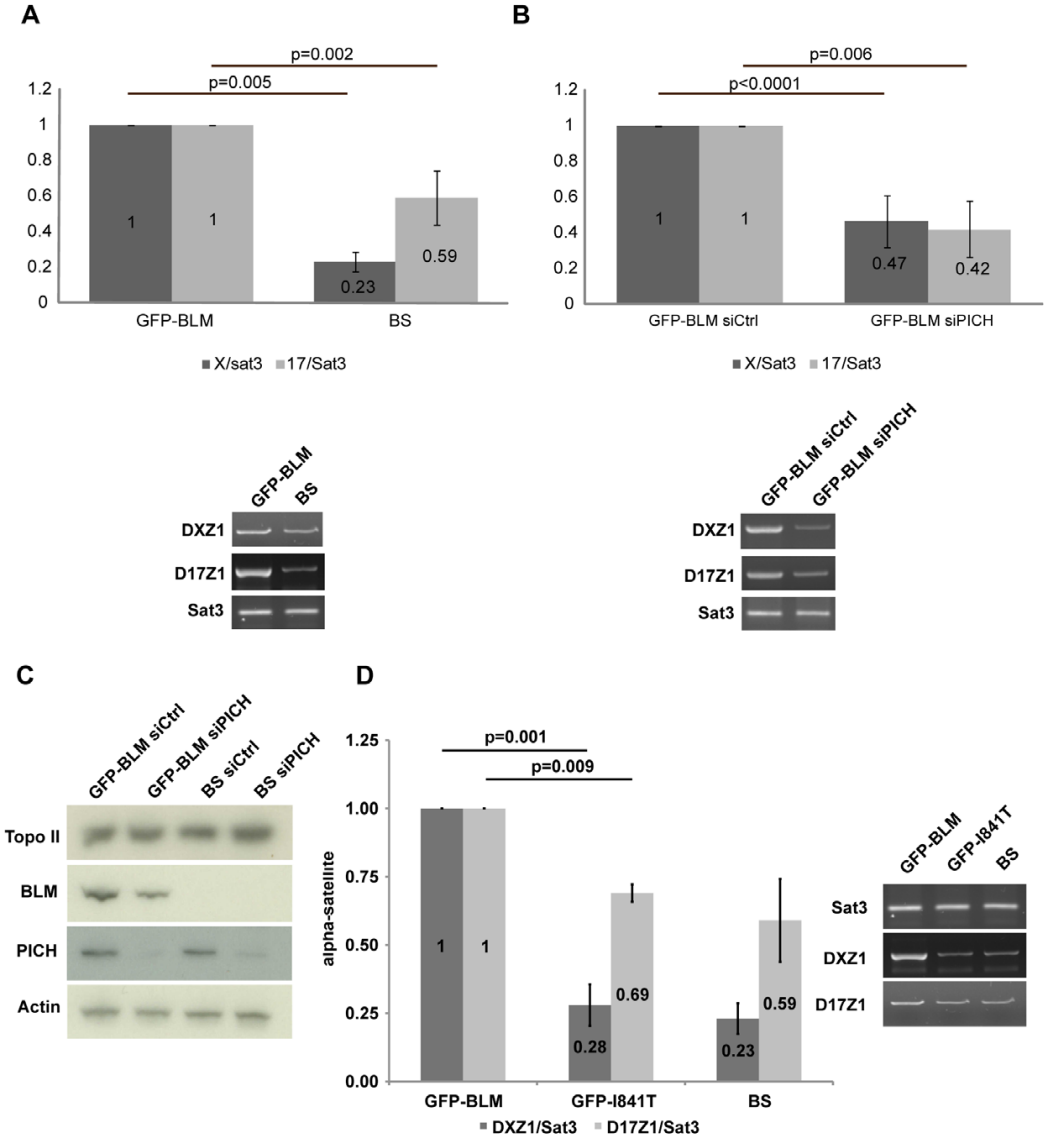
PICH and BLM are required for the recruitment of Topo IIα to centromeres (A) ChIP-PCR analysis of Topo IIα recruitment to pericentromeric region satellite 3 (Sat3) or to the centromeric DNA of chromosomes X (DXZ1) or 17 (D17Z1) was performed in GFP-BLM and BS cells (lower panels). The histogram shows relative enrichment in DXZ1 (DXZ1/Sat3) or D17Z1 (D17Z1/Sat3) sequences for active Topo IIα in GFP-BLM (defined as 1) and in BS cells (upper panels). The results presented are from three independent experiments. Error bars indicate the SD. (B) We performed the same experiments as in (A), with GFP-BLM cells transfected for 72 hours with control siRNAs or PICH siRNAs. (C) The amount of Topo II was evaluated in each cell line by western blotting. (D) We performed the same experiments as in (A), with GFP-BLM, GFP-I841T and BS cells.

Topo IIα is known to be responsible for the elimination of centromeric DNA catenation [16]. We investigated the possible role of BLM and PICH in centromeric decatenation, by assessing the recruitment of active Topo IIα to the pericentromeric region (satellite 3) and to the centromere of chromosomes X (α-satellite sequences DXZ1) and 17 (D17Z1). Topo IIα was trapped on DNA by 15 minutes of treatment with etoposide [18], and analyzed by chromatin immunoprecipitation (ChIP) coupled to semi-quantitative PCR (see Methods) in BS cells and GFP-BLM cells (Figure 5A) and in PICH-depleted GFP-BLM cells (siPICH) and their control cells (siCtrl) (Figure 5B). Immunoprecipitation of active Topo IIα in BS cells, PICH-depleted GFP-BLM cells and their respective control cells resulted in similar amplification of satellite 3 (Figure 5A and 5B, lower panels). We then used this as a control for normalization and found that the amounts of DXZ1 and D17Z1 amplified sequences were four and two times higher, respectively, in GFP-BLM control cells than in BS cells (Figure 5A, upper panel). DXZ1 and D17Z1 sequences bound to active Topo IIα were also twice as abundant in control cells as in PICH-depleted cells (Figure 5B, upper panel), whereas the total amount of Topo IIα was similar in all conditions (Figure 5C). We performed the same analysis with GFP-I841T cells, comparing the results obtained with those for BS and GFP-BLM cells, and obtained similar results for GFP-I841T cells and BS cells (Figure 5D). Thus, active Topo IIα is significantly less abundant at the centromeres of BLM-deficient cells and PICH-deficient cells than at those of control cells. These results also indicate that the helicase activity of BLM is involved in the recruitment of active Topo IIα to centromeres.

## Discussion

We report here a new localization and function for BLM at centromeres. We also report that BLM cooperates with PICH at centromeres, and that both these proteins play an important role in a previously unknown mechanism involving the recruitment of active Topo IIα to centromeres to eliminate DNA catenation before the onset of anaphase.

**Figure 6:**
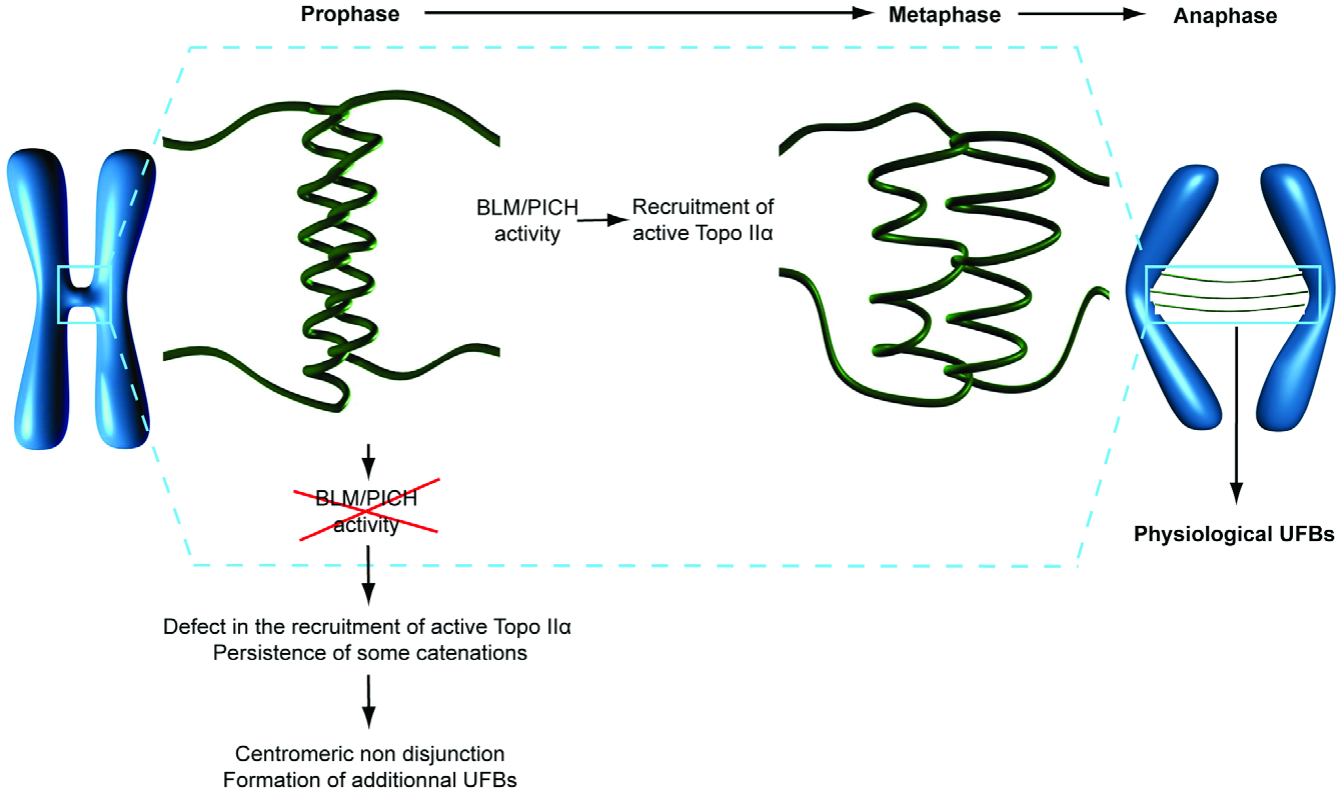
Model of PICH/BLM-dependent decatenation of centromeric DNA. We propose that the combined action of BLM and PICH promotes the organization of centromeric chromatin, thereby rendering some centromeric catenates accessible to Topo IIα. In the absence of BLM or PICH, a defect in the recruitment of active Topo IIα results in the persistence of some catenations leading to centromeric non disjunction and the formation of additional UFBs.

Our FISH experiments and EM images showed changes in the structure of centromeric chromatin in the absence of BLM or PICH (Figure 3). Recombinant PICH has recently been shown to have nucleosome remodeling activity *in vitro*, and BLM has been shown to be required for the chromatin remodeling function of PICH during anaphase *in vivo* [15]. On the other hand, changes in DNA topology are required for efficient mitotic decatenation by Top2, the fission yeast homolog of Topo IIα [19]. We therefore propose a model in which the combined action of BLM and PICH promotes the organization of centromeric chromatin, thereby rendering some centromeric catenates accessible to Topo IIα (Figure 6). This pathway may allow the Topo IIα-mediated resolution of some centromeric DNA catenations as soon as the cells enter prometaphase, thereby preventing centromeric non disjunction and the potential formation of additional UFBs that might interfere with abscission [7].

Our results also indicate that, in addition to their role in resolving physiological UFBs [5], [15], BLM and PICH may participate in the prevention of UFB formation. Thus, the additional centromeric UFBs that form in BLM-deficient cells or in cells treated with inhibitors of the catalytic activity of Topo IIα may include both newly formed UFBs and unresolved physiological UFBs: these UFBs are probably similar in nature but of different origins. Indeed, cohesin complexes preserve catenation at centromeres until the onset of anaphase, accounting for the presence of physiological UFBs [7]. The resolution of centromeric catenation therefore depends on cohesin removal [7]. However, our results indicate that some centromeric catenations are processed before the onset of anaphase to prevent the formation of additional UFBs. Thus, physiological UFBs may arise from catenations locally protected by cohesin complexes and processed at anaphase onset, whereas additional UFBs may arise from catenations not resolved at an earlier step.

In conclusion, the new centromeric decatenation mechanism reported here is probably the first step in a two-step centromeric decatenation process dependent on the coordinated action of BLM, PICH and Topo IIα. Moreover, our studies shed light on the mechanisms underlying the genetic instability and predisposition to cancer associated with Bloom’s syndrome.

## Materials and Methods

### Cell Lines, Cell Culture and Transfections

The GFP-BLM construct was kindly provided by Nathan Ellis. The SV40-transformed fibroblast cell line GM08505B, derived from the BS patient 42 (RaFr) of the Bloom’s syndrome registry, was obtained from the NIGMS Human Genetic Mutant Cell Repository (Camden, NJ, USA). BS and GFP-BLM cells were obtained by transfecting BS GM08505B cells with the EGFP-C1 vector alone (Clontech, Mountain View, CA), or with the same vector containing the full-length BLM cDNA [20], respectively, in the presence of JetPEI reagent (Ozyme). GFP-I841T cells were obtained by transfecting BS GM08505B cells as described above, with the EGFP-C1 vector containing the full-length BLM cDNA mutated at codon 841 (see site-directed mutagenesis).

HeLa and HeLa S3 cells, obtained from the American Tissue Culture Collection.

HeLaV cells and HeLashBLM cells were obtained by transfecting cells with an empty pSM2 vector or with the same vector encoding a short hairpin RNA sequence directed against BLM (Open Biosystems, clone V2HS-89234), respectively, with JetPEI reagent. After 48 h, selection with 1 to 5 µg/ml puromycin (Invivogen) was applied. Individual colonies were isolated and cultured in medium containing 0.5 µg/ml puromycin [10], [21].

All cell lines were cultured in DMEM (Gibco) supplemented with 10% decomplemented FCS (Invitrogen), L-glutamine and antibiotics.

Cells were transfected with siRNAs directed against BLM (48 h), PICH (72 h) and Rad21 (72 h) (ON-TARGETplus, SMARTpool, Dharmacon) and with a control non-targeting pool of siRNA (ON-TARGETplus siCONTROL Non Targeting Pool, Dharmacon) (100 nM final concentration) in the presence of Dharmafect 1 (Dharmacon) according to the manufacturer’s instructions. The sequences of all siRNA oligonucleotides used are presented in Table S1. The pool of siRNAs targeting PICH has been shown to have no effect on nocodazole-induced SAC (spindle assembly checkpoint) activation [22].

### Chemicals

Etoposide (Sigma) was used at a final concentration of 0.5 mM.

### Plasmid Construction and Site-directed Mutagenesis

Site-directed mutagenesis was performed with the EGFP-C1 vector containing the full-length BLM cDNA: Ile-841 and Gly-891 were mutated with the QuikChange XL site-directed mutagenesis kit (Agilent) according to the manufacturer’s instructions.

Primer 1 (5′-CCCAGGGTACAGAAGGACACCCTGACTCAGCTGAAG - 3′) and primer 2 (5′-CTTCAGCTGAGTCAGGGTGTCCTTCTGTACCCTGGG- 3′) were used to mutate Ile-841 and primer 3 (5′-GCACCACCCATATGATTCAGAGATAATTTACTGCCT- 3′) and primer 4 (5′-AGGCAGTAAATTATCTCTGAATCATATGGGTGGTG- 3′) were used to mutate Gly-891.

### Western-blot Analysis

Cells were lysed in 8 M urea buffer (8 M urea, 150 mM β-mercaptoethanol, and 50 mM Tris (pH 7.4) in water), sonicated and heated. Samples equivalent to 2.5 10^5^ cells were subjected to electrophoresis in NuPAGE Novex 4–12% Bis-Tris precast gels (Invitrogen). The procedures used for immunoblotting have been described elsewhere [23].

### Antibodies

All the commercial antibodies were used according to the manufacturers’ specifications. The primary antibodies were used for immunofluorescence (IF), immunoprecipitation (IP) or immunoblotting (IB) at the following concentrations: rabbit anti-BLM (1∶5000 for IB and 1∶200 for IF, AB476 from Abcam), goat anti-BLM (1∶150 for IF, C18 from Santa-Cruz Biotechnology), CREST serum (1∶500 for IF, from Antibody Incorporated (Fig. 1), kindly provided by I. Bahon-Riedinger (Fig. 2)), mouse anti-cyclin B1 (1∶500 for IF, sc-245 from Santa Cruz), mouse anti-Rad21 (1∶1000 for IB, 53A303 from Upstate), mouse anti-Topo II alpha (1∶1000 for IB and IF, 3D4 from Assay Design), mouse anti-Topo II alpha (1∶250 for IP, KiS1 from Millipore), anti-β-actin (1∶10000 for IB; Sigma), anti-GAPDH (1∶10000 for IB; Millipore), anti-PICH (1∶200 for IF and 1∶750 for IB, DO1P from Abnova) and anti-CENP-A (1∶200 for IF, AB13939 from Abcam). Secondary antibodies conjugated to Alexa Fluor 488, 555 and 633 (Molecular Probes, 1∶200-600) were used for immunofluorescence and secondary antibodies conjugated to horseradish peroxidase (Santa Cruz, 1∶5000-10000) were used for immunoblot detection.

### Reverse Transcription and Real-time Quantitative PCR

Total RNA was extracted with the RNeasy Mini kit (Qiagen) including a DNAse digestion step. cDNAs were synthesized with 250 ng of random hexamers (Invitrogen), 2 µg of RNA and Superscript II reverse transcriptase (Invitrogen). qPCR experiments were performed according to the MIQE Guidelines [24]. Amplification mixtures contained the cDNA template (1/100 dilution), SYBR Green Supermix 1× (BioRad) and 300 nM forward and reverse primers. Amplification was performed with the CFX96 detection system (BioRad). The primer sequences for Rad21 have been described on the website www.rtprimerdb.org (RTPrimerDB ID:8036). The relative quantities of the Rad21 cDNAs were normalized against two reference genes (RPL32, SDHA) chosen on the basis of their low M-value [25], [26].

### Immunofluorescence Microscopy

Immunofluorescence staining was performed as previously described [27] with the inclusion of a prepermeabilization incubation with 0.5% Triton X-100 before fixation for the detection of chromatid-bound protein. Nuclear DNA was detected by mounting slides in Prolong® Gold antifade reagent supplemented with DAPI (Invitrogen). Cell images were acquired with a 3-D deconvolution imaging system consisting of a Leica DM RXA microscope equipped with a piezoelectric translator (PIFOC; PI) placed at the base of a 63x PlanApo N.A. 1.4 objective, and a CoolSNAP HQ interline CCD camera (Photometrics). Stacks of conventional fluorescence images were collected automatically at a Z-distance of 0.2 µm (Metamorph software; Molecular Devices). Images are presented as maximum intensity projections generated with ImageJ software, from stacks deconvolved with an extension of Metamorph software [28].

### FISH Analysis

FISH was performed with the CEP-8 probe (CEN-8) from Abbott Vysis according to the manufacturer’s instructions with fixation of cells in paraformaldehyde. Images were acquired on a Leica SP5 confocal system, equipped with an argon laser, with 405 nm and 561 nm laser diodes, using a 63×/1.4 objective. All recordings were made with the appropriate sampling frequency (512×512 images, line average of 8 and zooming set to 8, pixel size: 60.2 nm). Image processing and quantification were carried out with the freely available Image J software [29]. FISH signal volume was determined with the 3D object counter plugin to Image J software [30]. A binary 3D image was generated, and a tagged map of all objects was obtained, based on pixel connexity (one tag per independent FISH signal, 26-neighbor connexity). The number of voxels (i.e. 3D pixels) carrying each tag was retrieved, then multiplied by the unit volume of the voxel (64.5 nm × 64.5 nm × 200 nm), to obtain the volume of each object. More information is available from the documentation supplied for the plugin: (http://imagejdocu.tudor.lu/lib/exe/fetch.php?media=plugin:analysis:3d_object_counter:3d-oc.pdf).

### Chromosome Spreading and Immunolabeling

Cells were left untreated or were transfected as indicated. After 48 h of transfection with an siRNA specific for Rad21, cells were transferred to slides in 6-well plates. After 24h at 37°C, colchicine was added to a final concentration of 0.1 µg/ml and the cells were incubated for 1 hour. The cells were then incubated in hypotonic solution (1∶5 (vol/vol) FCS-distilled water) and fixed by incubation with a 3∶1 (vol/vol) mixture of methanol and acetic acid. Cells were then stained by incubation with 2% Giemsa solution (VWR) for 16 minutes, rinsed in distilled water, dried, and mounted. Chromosomes were observed with a Leica DMRB microscope at 100× magnification. Metaphases were captured with a SONY DXC 930 P camera. Centromeric disjunction classification was evaluated by retrieving intensity profiles with the “plot profile function” of ImageJ applied to distance-calibrated images, along a line placed over the primary constriction region. For chromosome immunolabeling, chromosome spreads were obtained by cytocentrifugation and processed as previously described [27].

### Topo II ChIP Analysis

Cells (10^6^) were treated with 0.5 mM etoposide for 15 min. They were then lysed in 1.5 ml of buffer containing 1% Sarkosyl, 10 mM Tris-HCl (pH 7.5), 10 mM EDTA, and protease inhibitor mixture (Complete Mini, Roche) by 10 passages through a 21G needle. CsCl was added from a concentrated stock solution (7 M), to give a final concentration of 0.5 M. DNA was fragmented by sonicating lysates with a Bioruptor sonicator (Diagenode), with 20-second pulses (20 seconds on/20 seconds off), for 15 minutes at the high-power setting. Under these conditions, DNA fragments of 0.5 to 3 kb were generated, as shown by agarose gel electrophoresis. Before immunoprecipitation (IP), we added 3 volumes of a buffer containing 10 mM Tris-HCl (pH 7.5), 10 mM EDTA, 100 mM NaCl, 0.1% Triton X-100, and protease inhibitor mixture to 1 volume of lysate. The resulting mixture was then centrifuged for 15 min at 16 000 × g at 4°C. Preclearing was then achieved by adding 60 µl of Protein G plus agarose beads (Santa Cruz) and rotating the tubes for 2 h at 4°C. The unbound fraction recovered by centrifugation was used for IP reactions. It was incubated overnight, at 4°C, with gentle shaking, with 5 µg of specific anti-Topo II antibody (KiS1) or control antibody (mouse IgG). Protein G plus agarose beads (Santa Cruz) were added and the mixture was incubated for 1 h. The beads were collected by centrifugation and washed three times with 150 mM NaCl buffer (10 mM Tris-HCl pH 7.5, 10 mM EDTA, 150 mM NaCl, 0.1% Triton X-100, protease inhibitor). Three rounds of elution were then carried out with 500 mM NaCl buffer (10 mM Tris-HCl pH 7.5, 10 mM EDTA, 500 mM NaCl, 0.1% Triton X-100, protease inhibitor), with the beads collected by centrifugation between elutions. The DNA fragments bound to the beads were treated with 50 µg/ml RNase A, at 55°C, for 30 min, then with 200 µg/ml proteinase K at 55°C overnight. They were then purified with the MiniElute PCR Purification kit (QIAGEN), according to the manufacturers’ instructions. DNA fragments were quantified with the Quant-IT Pico Green dsDNA kit (InvitrogenTM, Molecular Probes®), according to the manufacturer’s protocol. Alpha-satellite DNA (20 pg) from chromosome X or chromosome 17 (DXZ1 and D17Z1, respectively) [31] or from pericentromeric DNA satellite 3 [32] was amplified by semiquantitative PCR. PCR products were analyzed by agarose gel electrophoresis and quantified with Image J (NIH) software. The signal obtained from the GFP-BLM cells was taken as 1.

### Electron Microscopy

BS and GFP-BLM cells grown at low density on coverslips were fixed by incubation with 2% paraformaldehyde, 2.5% glutaraldehyde in 0.1 M cacodylate buffer for 24 h and postfixed by incubation with 1% (wt/vol) OsO_4_ supplemented with 1.5% (wt/vol) ferrocyanide. They were then dehydrated in ethanol and embedded in Epon. Ultrathin sections were prepared with a Reichert UltracutS ultramicrotome (Leica) and viewed with a TEM CM120 Philips electron microscope after counterstaining with uranyl acetate and lead citrate.

### Statistical Methods

Significance was assessed with Student’s *t*-test. For all tests, p<0.05 was considered statistically significant.

## Supporting Information

Table S1 **Sequences of the siRNAs used in this study.**
(XLS)

Figure S1 **GFP-BLM localizes to the PML body.** Colocalization of GFP-BLM (green) with PML (red). Nuclei were visualized by DAPI staining (blue). Scale bar = 5 µm.
(TIF)

Figure S2 **Kinetochore structure is not profoundly affected in BLM-deficient cells.** CENP-A immunostaining of BS and GFP-BLM metaphase cells revealed a similar localization in the two types of cells. Chromosomes are visualized by DAPI staining (red). Scale bar = 5 µm. Similar results were obtained with 5 representative kinetochore/centromere proteins (AURORA B, CENP-I, HEC, BUB1 and BUBR1).
(TIF)

Figure S3 **Structural defects at the centromeres in BLM- and PICH-deficient HeLa cells.** Comparison of the volume of the centromeric FISH signal detected on chromosomes 8 from HeLaV-siCtrl (defined as 1), HeLa V-siPICH, HeLash-siBLM and HeLash-siBLM-siPICH cells (right panel). We analyzed between 18 and 27 metaphase cells from two independent experiments for each cell line. BLM and PICH protein levels were assessed by immunoblotting, probing the same membrane with anti-BLM (ab-476) and anti-PICH antibodies and with anti-β actin antibody, as a loading control (upper left panel).
(TIF)

Figure S4 **BLM-downregulated and PICH-downregulated HeLa cells display non disjunction of centromeres.** HeLa cells were transfected for 72 hours with Rad21 siRNAs and transfected with control siRNAs, BLM siRNAs or PICH siRNAs. BLM, PICH and Rad21 protein levels were assessed by immunoblotting, probing the same membrane with anti-BLM (ab-476), anti-PICH and anti-Rad21 antibodies and with anti-β actin antibody, as a loading control (left panel). Chromosome spreads were performed and sorted on the basis of their phenotype: X-shapes, incomplete disjunction or complete disjunction. We analyzed 500 spreads from two independent experiments for each cell line. The frequency of each phenotype, in each of the three cell lines, is shown in the histogram (right panel). Bars represent SD.
(TIF)

Figure S5 **siRNA-mediated Rad21 downregulation was similarly effective in all conditions.** GFP-BLM or HeLa cells were transfected for 72 hours with the indicated siRNAs. Rad21 mRNA levels were determined by reverse transcription quantitative PCR in all conditions. Histograms represent the amplification of Rad21 mRNA from one experiment in triplicate for GFP-BLM and BS cells and are the mean of the amplification of Rad21 mRNA in triplicate in two independent experiments for HeLa cells. Bars represent s.e.m.
(TIF)

Figure S6 **Centromeric UFBs are not detectable in PICH-deficient cells.** (A) (Upper panels) GFP-BLM cells were transfected for 72 hours with PICH or control siRNAs. UFBs were detected by immunostaining of BLM and PICH and quantified (164 UFBs from 129 siCtrl cells and 21 UFBs from 129 siPICH cells were scored in three independent experiments, respectively). Bars represent SDs. (Lower panel) Representative example of a UFB in anaphase cells revealed by BLM staining. The nucleus was visualized by DAPI staining (blue). Scale bar = 5 µm. (B) Total number of UFBs detected and scored in PICH-downregulated cells. UFBs positive for PICH only or for BLM only or for both PICH and BLM were scored in three independent experiments including a total of 129 cells transfected with control siRNAs and 129 cells transfected with PICH siRNAs.
(TIF)
