# Supplementary figures and images for: Bloom’s Syndrome and PICH Helicases Cooperate with Topoisomerase IIα in Centromere Disjunction before Anaphase
Source: PLoS One. 2012 Apr 26;7(4):e33905. doi: 10.1371/journal.pone.0033905 (PMC3338505; doi:10.1371/journal.pone.0033905)

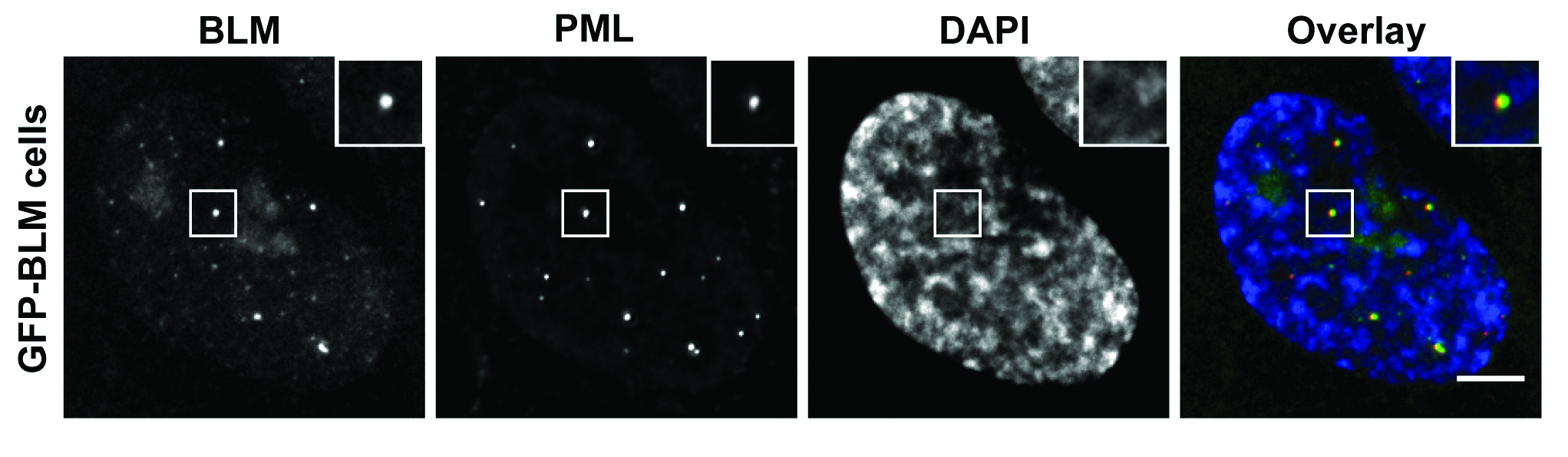

Supplement: Figure S1 — GFP-BLM localizes to the PML body. Colocalization of GFP-BLM (green) with PML (red). Nuclei were visualized by DAPI staining (blue). Scale bar = 5 µm. (TIF) [file pone.0033905.s002.tif]

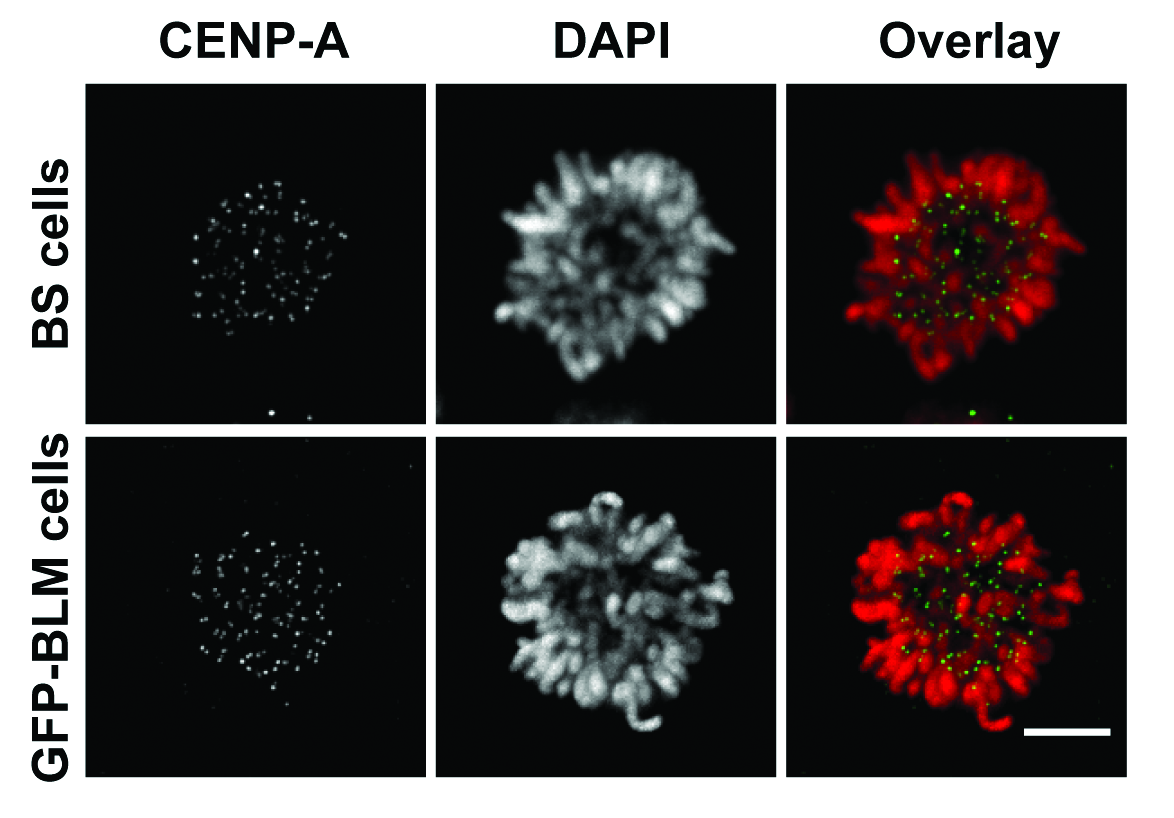

Supplement: Figure S2 — Kinetochore structure is not profoundly affected in BLM-deficient cells. CENP-A immunostaining of BS and GFP-BLM metaphase cells revealed a similar localization in the two types of cells. Chromosomes are visualized by DAPI staining (red). Scale bar = 5 µm. Similar results were obtained with 5 representative kinetochore/centromere proteins (AURORA B, CENP-I, HEC, BUB1 and BUBR1). (TIF) [file pone.0033905.s003.tif]

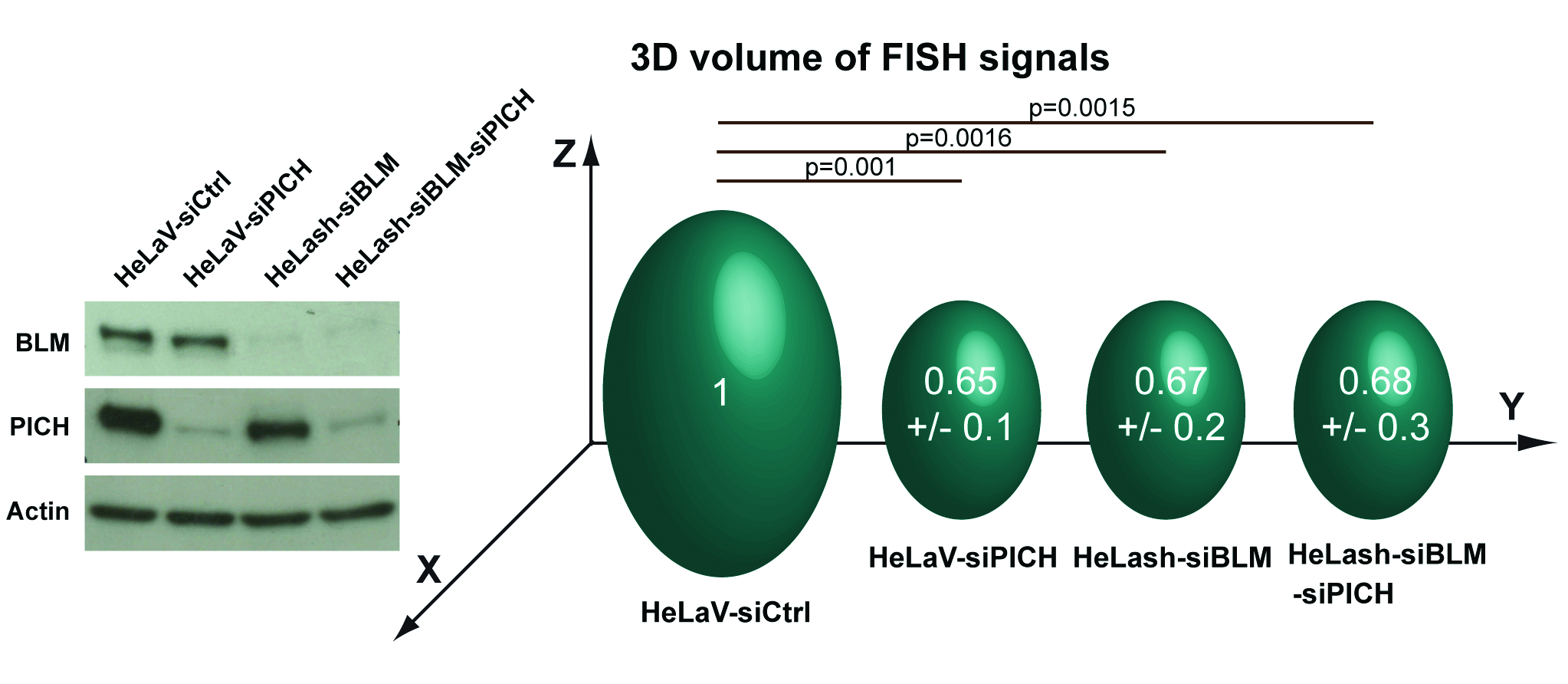

Supplement: Figure S3 — Structural defects at the centromeres in BLM- and PICH-deficient HeLa cells. Comparison of the volume of the centromeric FISH signal detected on chromosomes 8 from HeLaV-siCtrl (defined as 1), HeLa V-siPICH, HeLash-siBLM and HeLash-siBLM-siPICH cells (right panel). We analyzed between 18 and 27 metaphase cells from two independent experiments for each cell line. BLM and PICH protein levels were assessed by immunoblotting, probing the same membrane with anti-BLM (ab-476) and anti-PICH antibodies and with anti-β actin antibody, as a loading control (upper left panel). (TIF) [file pone.0033905.s004.tif]

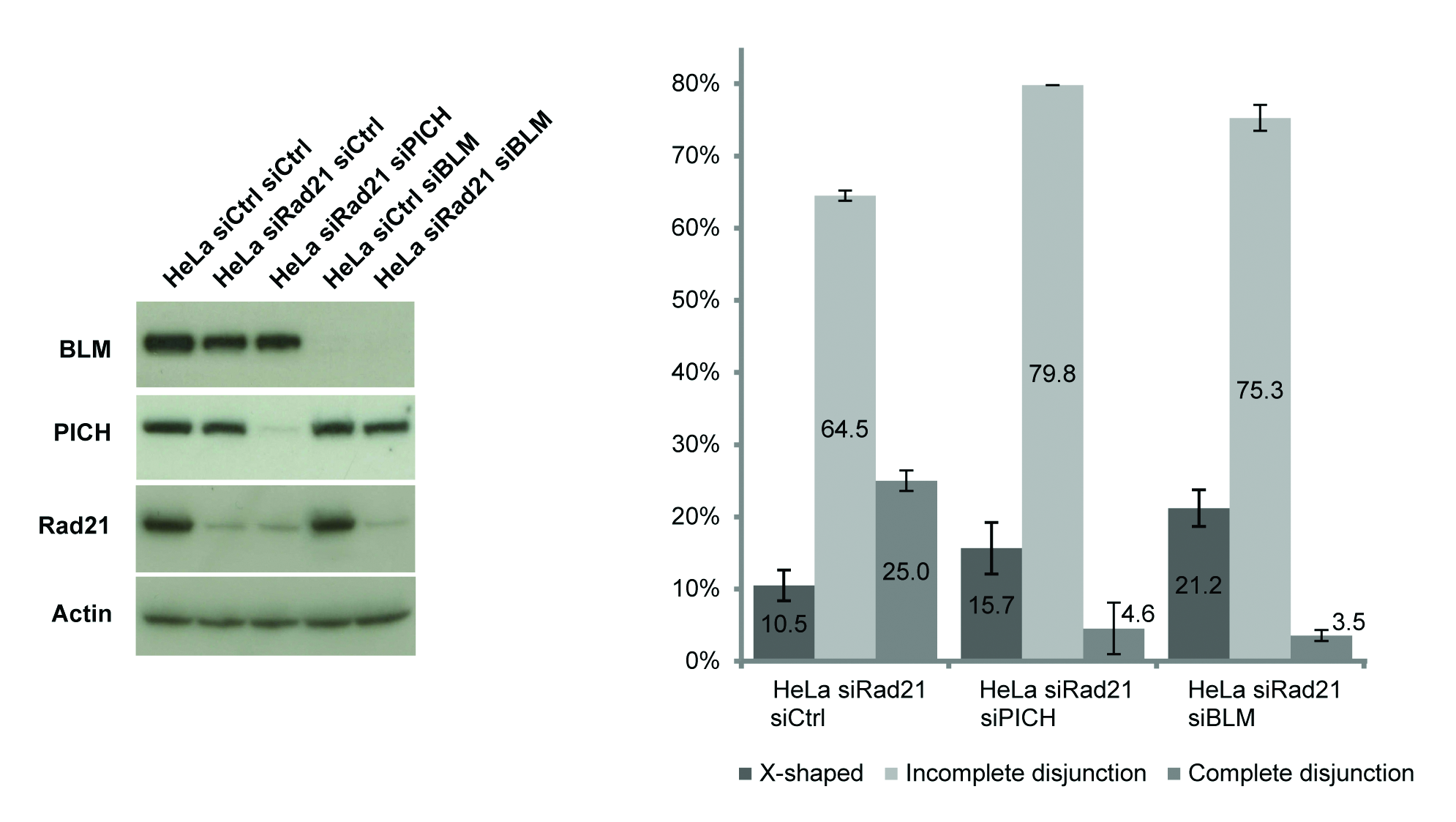

Supplement: Figure S4 — BLM-downregulated and PICH-downregulated HeLa cells display non disjunction of centromeres. HeLa cells were transfected for 72 hours with Rad21 siRNAs and transfected with control siRNAs, BLM siRNAs or PICH siRNAs. BLM, PICH and Rad21 protein levels were assessed by immunoblotting, probing the same membrane with anti-BLM (ab-476), anti-PICH and anti-Rad21 antibodies and with anti-β actin antibody, as a loading control (left panel). Chromosome spreads were performed and sorted on the basis of their phenotype: X-shapes, incomplete disjunction or complete disjunction. We analyzed 500 spreads from two independent experiments for each cell line. The frequency of each phenotype, in each of the three cell lines, is shown in the histogram (right panel). Bars represent SD. (TIF) [file pone.0033905.s005.tif]

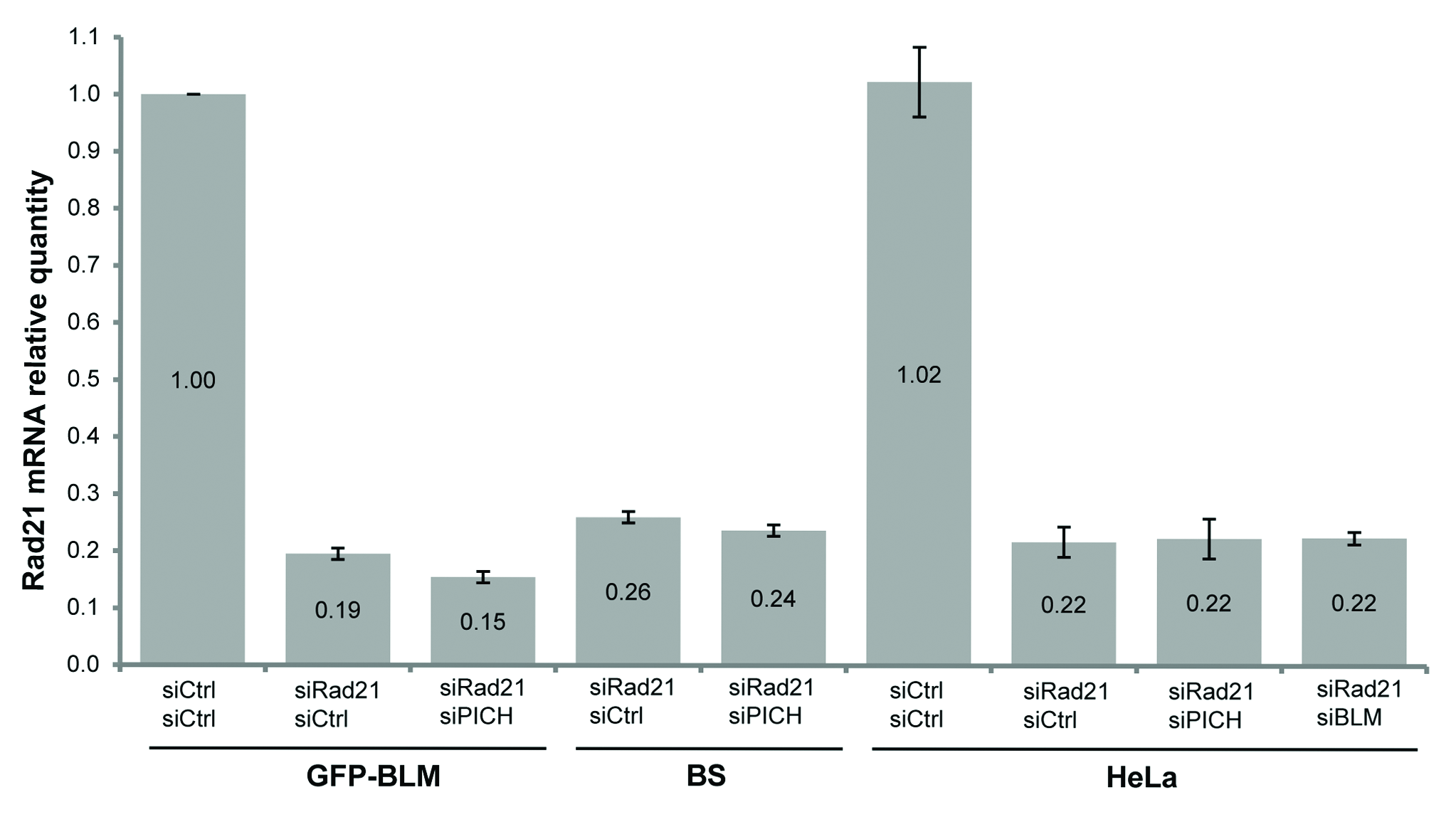

Supplement: Figure S5 — siRNA-mediated Rad21 downregulation was similarly effective in all conditions. GFP-BLM or HeLa cells were transfected for 72 hours with the indicated siRNAs. Rad21 mRNA levels were determined by reverse transcription quantitative PCR in all conditions. Histograms represent the amplification of Rad21 mRNA from one experiment in triplicate for GFP-BLM and BS cells and are the mean of the amplification of Rad21 mRNA in triplicate in two independent experiments for HeLa cells. Bars represent s.e.m. (TIF) [file pone.0033905.s006.tif]

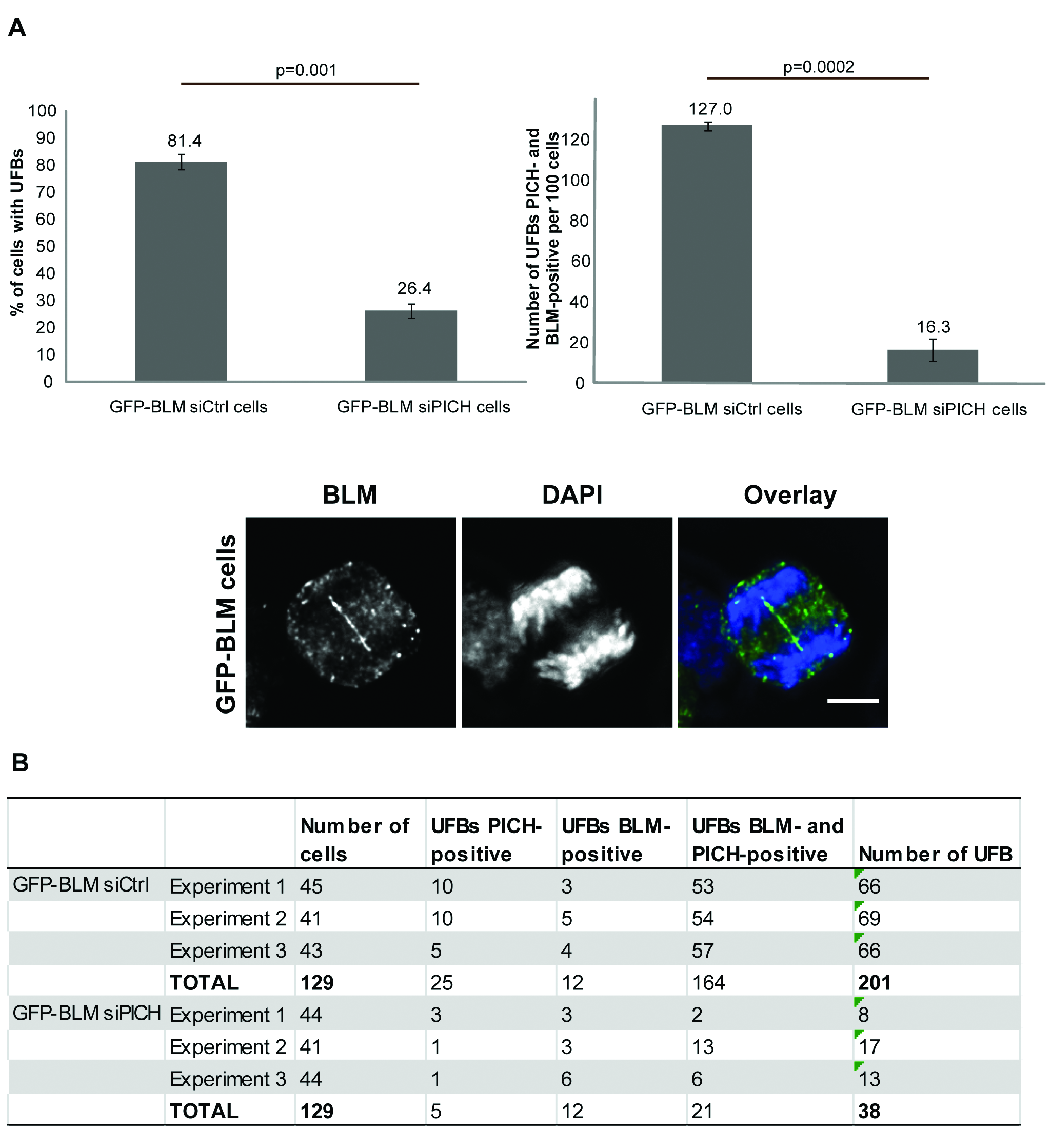

Supplement: Figure S6 — Centromeric UFBs are not detectable in PICH-deficient cells. (A) (Upper panels) GFP-BLM cells were transfected for 72 hours with PICH or control siRNAs. UFBs were detected by immunostaining of BLM and PICH and quantified (164 UFBs from 129 siCtrl cells and 21 UFBs from 129 siPICH cells were scored in three independent experiments, respectively). Bars represent SDs. (Lower panel) Representative example of a UFB in anaphase cells revealed by BLM staining. The nucleus was visualized by DAPI staining (blue). Scale bar = 5 µm. (B) Total number of UFBs detected and scored in PICH-downregulated cells. UFBs positive for PICH only or for BLM only or for both PICH and BLM were scored in three independent experiments including a total of 129 cells transfected with control siRNAs and 129 cells transfected with PICH siRNAs. (TIF) [file pone.0033905.s007.tif]
